# Draft Genome Sequence of Weissella paramesenteroides STCH-BD1, Isolated from Ensiled Sorghum bicolor

**DOI:** 10.1128/MRA.01328-20

**Published:** 2021-02-18

**Authors:** Ashley V. Baugh, Thomas M. Howarth, Katrina L. West, Lydia E. J. Kerr, John Love, David A. Parker, Jeffrey R. Fedenko, Richard K. Tennant

**Affiliations:** a Shell International Exploration and Production, Inc., Biodomain, Shell Technology Centre Houston, Houston, Texas, USA; b Biosciences, University of Exeter, Exeter, Devon, United Kingdom; University of Southern California

## Abstract

Weissella paramesenteroides has potential as an industrial biocatalyst due to its ability to produce lactic acid. A novel strain of *W. paramesenteroides* was isolated from ensiled sorghum. The genome was sequenced using a hybrid assembly of Oxford Nanopore and Illumina data to produce a 2-Mbp genome and 22-kbp plasmid sequence.

## ANNOUNCEMENT

*Weissella* spp. are Gram-positive coccobacillus-shaped bacteria (1, 2) which were reclassified from the genus *Leuconostoc* (2). *Weissella* spp. have received industrial interest due to their probiotic nature and ability to ferment a range of carbohydrates to lactic and acetic acids (3). Specifically, Weissella paramesenteroides is able to produce d-lactic acid (2, 4) and is commonly identified in silage material (5).

Weissella paramesenteroides STCH-BD1 was isolated from ensiled Sorghum bicolor. Fresh sorghum, cultivated in Florida, was ensiled for 180 days at approximately 21°C in 5-gallon buckets fitted with a 3-piece airlock to maintain anaerobic conditions. Ensiled sorghum was squeezed using a garlic press, and the pressate was spread onto an MRS agar plate (6), which was incubated aerobically at 30°C. A single colony of the isolated bacteria was cultured in MRS broth and incubated aerobically at 30°C and 180 rpm for 16 h. Cells were lysed in lysis tubes containing lysing matrix E (MP Bio, USA) and were placed in the MP Bio FastPrep instrument and operated at 6.0 ms^−1^ for 40 s. Lysates were centrifuged, and DNA was purified using the GeneJET genomic DNA purification kit (Thermo Scientific, USA). Oxford Nanopore Technologies (UK) libraries were prepared using the SQK-RBK004 rapid sequencing kit and sequenced on a MinION instrument attached to a MinIT device (Oxford Nanopore Technologies) using an R9.4a flow cell (Oxford Nanopore Technologies). Oxford Nanopore sequence reads were base called using Guppy v4.2.2 operating in high-accuracy mode and yielded 398,617 DNA sequence reads with an *N*_50_ value of 3,091 bp. Illumina DNA sequencing libraries were prepared using the Nextera XT library preparation kit (Illumina, USA) and sequenced on the Illumina MiSeq platform, using a 250-bp paired-end sequencing flowcell which yielded 273,888 DNA sequence reads. Default parameters were used for subsequent analysis except where otherwise noted. A *de novo* hybrid assembly using the raw Illumina and Oxford Nanopore reads was performed using MaSuRCA v3.4.2 (7) which was configured as part of the pipeline to use Flye (8) as the final assembler of the corrected reads.

**FIG 1:**
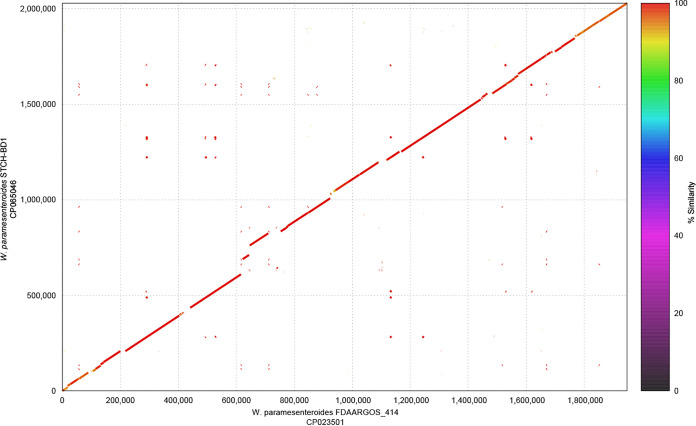
Genome alignment between Weissella paramesenteroides STCH-BD1 (GenBank accession number CP065046) and Weissella paramesenteroides FDAARGOS_414 (CP023501) chromosomes. Percentage similarities are displayed using a rainbow color scale.

The genome sequence was assembled to a single, linear 2,052,436-bp contig with a GC content of 38% and 210-fold coverage. A circular 22,825-bp contig with a GC content of 33% and 870-fold coverage was identified by Flye and designated a plasmid. The assembled genome was verified using BWA-MEM v0.7.17 (9) and validated in Tablet v1.19.09.03 (10) to ensure complete coverage. Taxonomic classification of the isolate was performed using Kraken 2 v2.0.7 (11) against the standard bacterial database, and fragments of the completed genome were taxonomically verified using NCBI blastn (12). The assembled *W. paramesenteroides* STCH-BD 1 genome was annotated using the NCBI Prokaryotic Genome Annotation Pipeline (13), which identified 1,934 coding sequences. The completed genome of *W. paramesenteroides* FDAARGOS_414 which was available from NCBI, under accession number CP023501, was compared with the genome of *W. paramesenteroides* STCH-BD 1 using the dnadiff package v1.3 within the MUMmer package v4.0.0rc1 (14) and visualized using mummerplot with the --color option (Fig. 1). The two genomes of *W. paramesenteroides* shared 98.7% homology between the two chromosomes.

### Data availability.

The draft genome sequence of *W. paramesenteroides* STCH-BD1 is deposited in GenBank under the accession numbers CP065045 and CP065046. Oxford Nanopore and Illumina DNA sequence reads have been deposited in the NCBI Sequence Read Archive under accession numbers SRR13083241 and SRR13083242, respectively.
